# Statistical Methods for Detecting Nonlinear Relationships in Gene Expression and Omics Data: A Review

**DOI:** 10.3390/ijms27135700

**Published:** 2026-06-24

**Authors:** Łukasz Huminiecki

**Affiliations:** Institute of Computer Science, Faculty of Natural Sciences, University of Siedlce, 08-110 Siedlce, Poland; lukasz.huminiecki@uws.edu.pl

**Keywords:** nonlinear dependence, nonlinear correlation, mutual information, Chatterjee correlation, maximal information coefficient, gene expression analysis, omics data analysis, gene regulatory networks, bioinformatics

## Abstract

High-throughput technologies such as RNA-seq and single-cell transcriptomics generate increasingly large and high-dimensional gene expression datasets in which nonlinear dependence structures are common. Because classical methods primarily capture linear associations, they may fail to characterize many biologically relevant patterns of dependence. To address this limitation, diverse nonlinear dependence measures—including information-theoretic, rank-based, kernel-based, distance-based, copula-based, and clustering-based approaches—have been developed. However, the field remains fragmented, and comparative evaluations are often inconsistent. This review organizes nonlinear methods into major methodological families and critically compares their statistical behavior, strengths, limitations, and characteristic modes of failure. We emphasize that method selection depends on matching inferential objectives to estimator assumptions, analytical constraints, and characteristic failure modes. By identifying recurring trade-offs among flexibility, robustness, interpretability, and computational scalability, we provide scenario-based guidance for method selection in transcriptomics, network inference, and functional genomics. In doing so, we aim to align inferential objectives with analytical requirements, supporting principled and application-specific use of nonlinear dependence methods in modern omics research.

## 1. Introduction

High-throughput omics technologies have revealed complex and often nonlinear relationships in gene regulation, signaling, and cellular states [1]. Yet classical linear measures, such as Pearson correlation, often fail to characterize these dependencies. To address this limitation, diverse nonlinear dependence frameworks have been developed, including information-theoretic [2], rank-based [3,4,5,6], kernel-based [7,8,9,10], distance-based [11], copula-based [12], and clustering-based approaches [13]. These methods differ not only in how they define dependence, but also in the inferential objectives they prioritize, including equitability, robustness, scalability, interpretability, and sensitivity to specific forms of biological structure [3,14,15]. Consequently, no single method is universally optimal.

Because different methods prioritize different inferential objectives, practical guidance on method selection remains fragmented. Existing reviews often focus on individual method families or enumerate methods without providing a comparative perspective. In contrast, this review provides a critical synthesis that avoids privileging any single measure. Both emerging and established methods are compared with respect to their assumptions, strengths, limitations, and characteristic modes of failure.

Moreover, existing reviews capture only parts of the nonlinear dependence landscape. General statistical surveys emphasize dependence theory without focusing on omics applications and largely predate Xi, multimodal integration, and modern single-cell inference frameworks [16]. More recent studies centered on Xi and related rank-based statistics primarily examine the mathematical properties of individual estimators rather than comparative bioinformatics workflows [3,6,17,18]. Conversely, reviews of gene regulatory networks and single-cell inference focus mainly on graph learning and systems biology pipelines rather than nonlinear dependence methodology itself [19]. Method-centered studies—including foundational papers on maximal information coefficient (MIC)—were designed to develop or compare individual estimators under selected statistical settings rather than to synthesize nonlinear inference across contemporary omics applications [2,15]. In contrast, the present review integrates diverse approaches within a unified inferential framework and compares established and emerging methods, with particular emphasis on developments from the past five years.

Rather than cataloging methods individually, we focus on the inferential trade-offs that distinguish them, including differences arising from data structure, sample size, dimensionality, and noise. As omics analysis advances—toward multimodal integration, graph-based inference, and single-cell atlases—these trade-offs become critical [20,21,22]. Misaligned choices can distort network inference and functional interpretation.

To ensure practical relevance, we conclude with scenario-based decision frameworks. These provide actionable guidance for selecting methods in specific omics scenarios—from transcriptomic screening to time-series analysis and gene regulatory network inference. By synthesizing the field’s methods through a lens of critical comparison, we align statistical tools with biological complexity—providing principled, nonredundant method selection for modern molecular data analysis.

Supplementary Table S1 places major nonlinear dependence frameworks within their historical and contemporary methodological context. Beyond summarizing foundational developments, it contrasts established, mature, active, and emerging approaches with respect to their adoption in omics research, current research activity, and principal areas of methodological innovation.

## 2. Conceptual Framework for Nonlinear Association in Bioinformatics

### 2.1. Information-Theoretic Dependence Measures

Information-theoretic methods quantify dependence through shared information rather than covariance-based association.

#### 2.1.1. Mutual Information (MI)

MI is the foundational information-theoretic measure of statistical dependence. Introduced by Shannon [23], it quantifies the reduction in uncertainty about one variable provided by knowledge of another and is capable of detecting arbitrary linear and nonlinear associations. MI does not assume a particular functional form and has been widely applied in transcriptomics, gene regulatory network inference, and systems biology [24]. However, practical application of MI depends critically on accurate probability-density estimation, making performance sensitive to sample size, dimensionality, discretization strategy, and noise structure [14].

#### 2.1.2. MIC

To address some of these challenges, Reshef et al. introduced MIC [2]. By maximizing normalized mutual information across alternative grid partitions, MIC placed relationships with distinct geometries on a common scale. Its central contribution was approximate equitability: associations were ranked according to noise level rather than functional form, extending dependence analysis beyond linear and monotonic structure.

In omics applications, this enabled systematic exploration of regulatory relationships without requiring prior assumptions regarding functional form. Consequently, MIC emerged as one of the most influential nonlinear dependence measures in molecular data analysis, stimulating extensive methodological development, and application across diverse omics domains [2,14,15,25].

The flexibility required for equitability, however, increased estimator variance and computational burden in sparse high-dimensional settings (Section 3.2). Subsequent variants therefore prioritized statistical stability and testing power. MIC estimator (MICe) improved finite-sample consistency, whereas total Information Coefficient estimator (TICe) increased sensitivity by aggregating information across partition structures instead of optimizing a single partition score [15].

#### 2.1.3. Beyond Pairwise Association

Early nonlinear dependence analysis focused primarily on isolated pairwise relationships. Subsequent extensions of MIC therefore shifted toward interaction-aware inference.

Approaches such as MIC(X_1_; X_2_; Y) estimated whether combinations of predictors jointly contributed information about a biological outcome [26]. In cancer transcriptomics, these methods identified synergistic gene interactions that were weak individually but highly informative collectively, extending nonlinear dependence analysis toward multivariate systems inference. Increasing dimensionality amplified computational complexity and estimator instability (Section 3.2).

#### 2.1.4. From Equitability to High-Dimensional Screening

Equitability and independence testing represent distinct inferential objectives. Equitable measures seek to characterize and compare the strength of different relationships, whereas independence tests focus on determining whether any statistically significant dependence is present. Consequently, methods optimized for independence testing often sacrifice equitable ranking in favor of greater detection power.

The development of the Total Information Coefficient (TIC) reflected growing recognition that exploratory ranking and statistical detection are fundamentally different inferential tasks [15]. Whereas MIC emphasized equitable comparison across associations with distinct geometries, TIC prioritized statistical power for independence testing in large-scale screening settings by aggregating information across partition structures.

This distinction became increasingly relevant in large-scale screening settings. In such contexts, the primary challenge is often not interpretability of individual relationships, but reliable detection under severe multiple-testing burden. TIC therefore shifted nonlinear dependence analysis toward scalable screening architectures optimized for high-dimensional molecular discovery.

The resulting workflows increasingly separated dependence filtering from downstream ranking and interpretation. This modularization reflected a broader transition within computational biology from standalone statistical estimators toward staged inferential workflows integrating feature screening, representation learning, graph construction, and multimodal data integration to support omics-scale analysis [15,20,27,28].

#### 2.1.5. Scalable Approximation and Infrastructure-Aware Inference

Later refinements of MIC-derived methods focused less on expanding conceptual flexibility and more on adapting nonlinear inference to large-scale omics computation. Methods such as ChiMIC [29], BackMIC [30], and UIC [31] simplified or constrained adaptive partition optimization to improve computational efficiency.

### 2.2. Rank-Based Dependence Measures

Rank-based methods evaluate dependence through ordinal structure rather than explicit reconstruction of relationship geometry [3,6,17]. Rather than modeling the precise form of an association, these methods assess whether the relative ordering of observations contains systematic evidence of dependence. In essence, they evaluate whether observations that rank similarly with respect to one variable also exhibit consistent rank structure with respect to another.

#### Chatterjee’s Coefficient of Correlation

Chatterjee’s coefficient of correlation (Xi) exemplifies the growing emphasis on scalable rank-based inference for nonlinear dependence analysis [3]. Xi quantifies the extent to which knowledge of one variable reduces uncertainty in the rank ordering of another. Consequently, it is sensitive to a broader range of dependence structures while retaining the ordinal inference framework characteristic of rank-based methods [3].

The importance of Xi lies in its formulation of dependence through rank-order predictability rather than explicit reconstruction of relationship geometry. Whereas methods such as MIC devote substantial computation to reconstructing relationship patterns, Xi focuses on whether the ordering of observations itself contains evidence of dependence. This simplification enables efficient screening of millions of candidate associations in large-scale transcriptomic and single-cell datasets while retaining sensitivity to diverse nonlinear relationships.

Applications in oscillatory transcriptomics illustrate this property. Xi identified cyclic transcriptional structure in yeast cell-cycle datasets that conventional correlation methods often failed to detect [3]. Importantly, this sensitivity emerged without explicit modeling of periodicity, instead reflecting the ability of rank-based methods to capture repeated reorganization across temporal progression.

Rank-based approaches are sensitive to changes in the relative organization of samples rather than relying solely on a single global association pattern. For example, regulatory relationships may strengthen, weaken, or reverse across cellular states, developmental stages, or disease conditions, even when no simple dependence structure is apparent across the dataset as a whole.

Consider a developmental trajectory in which cells progress from progenitor to differentiated states. A target gene need not increase linearly with a regulator; instead, cells exhibiting relatively high regulator expression may consistently occupy higher positions in the target-gene ranking. Xi captures this ordering information without requiring reconstruction of the precise functional form linking the two genes.

### 2.3. Kernel-Based Dependence Measures

Kernel-based methods measure statistical dependence through similarity among samples rather than through shared information, covariance, or rank order. Instead of asking whether variables change together, they ask whether samples that are similar with respect to one variable are also similar with respect to another [32].

Once dependence is formulated in terms of similarity, the central question becomes how to compare similarity patterns across variables. Kernel-based methods address this problem by converting observations into similarity representations and then quantifying the extent to which these representations agree. This idea underlies kernelized correlation, the Hilbert–Schmidt Independence Criterion (HSIC), and related reproducing kernel Hilbert space (RKHS)-based approaches.

#### Kernelized Correlation and RKHS-Based Inference

Kernel-based methods operationalize dependence by comparing similarity structures induced by different variables. For each variable, a kernel function is used to quantify pairwise similarity among samples, producing a kernel matrix that summarizes how observations relate to one another. Dependence is then assessed by evaluating the extent to which the similarity structure generated by one variable agrees with that generated by another. Consequently, samples that are similar with respect to one molecular feature and also similar with respect to another contribute positively to the estimated dependence.

Methods such as HSIC implement this principle within the framework of RKHS, which provide a mathematically convenient representation of similarity relationships among observations [32,33]. Rather than measuring covariance between the original variables, HSIC effectively measures agreement between their kernel-induced similarity structures. Kernelized correlation (Kc) follows a related strategy by estimating association after nonlinear transformation into RKHS feature spaces, enabling detection of broad classes of nonlinear relationships without specification of explicit functional models [7].

Recent developments have focused on conditional dependence testing, feature-selection frameworks such as HSIC-Lasso, and statistically calibrated inference in large-scale settings, making kernel-based methods one of the most active areas of nonlinear dependence research in contemporary omics analysis [8,9,10].

### 2.4. Distance Correlation and Energy-Based Dependence

Distance correlation (dCor) and related energy statistics provide a general framework for nonlinear dependence estimation based on pairwise distances between observations rather than explicit functional reconstruction [34,35]. Distance correlation equals zero if and only if independence holds, giving the method a strong theoretical characterization of general dependence.

Conceptually, distance-based approaches occupy an intermediate position between information-theoretic and kernel-based inference. Rather than estimating shared information or embedding variables into reproducing kernel Hilbert spaces, dCor quantifies whether geometric distance relationships among observations are preserved across variables. This formulation allows detection of broad classes of nonlinear and non-monotonic dependence while remaining nonparametric and comparatively interpretable.

Distance correlation proved particularly attractive in omics analysis because it naturally extends to multivariate random vectors and does not require specification of functional form. Applications include transcriptomic feature screening, multimodal association analysis, and high-dimensional independence testing. Recent research has focused primarily on asymptotic behavior, estimator refinement, and statistical testing theory in high-dimensional settings rather than on new dependence formulations [11,36,37,38]. However, distance-based methods also exhibit important limitations. Distance concentration and dimensional scaling can affect estimator behavior and testing power, motivating modified statistics, regularization strategies, robust estimators, and asymptotic corrections [36,39].

### 2.5. Copula-Based Dependence Modeling

Copula-based methods separate marginal distributions from dependence structure [12]. Recent omics applications increasingly employ Gaussian copula and semiparametric copula frameworks for transcriptomic network inference, single-cell multi-omics integration, and large-scale co-expression analysis [40,41]. These methods proved particularly useful for integrating heterogeneous molecular measurements, including RNA-seq, chromatin accessibility, DNA methylation, and microbiome profiles, where conventional correlation estimators are sensitive to non-normal marginals and compositional effects. Copula-based approaches are also increasingly incorporated into graphical modeling and multimodal network reconstruction workflows for systems-level omics inference [42]. However, flexible copula formulations may introduce substantial computational complexity in large-scale settings, motivating ongoing work on sparse estimation, regularization, and scalable semiparametric inference for large-scale molecular datasets.

### 2.6. Clustering-Based and Heterogeneity-Aware Dependence

Clustering-based methods evaluate dependence through concordance of sample partitions and are particularly relevant when dependence structure varies across subpopulations (Section 3.4).

#### Clustermatch Correlation Coefficient (CCC)

CCC represents a clustering-based approach to dependence estimation [13]. CCC quantifies agreement between sample partitions induced by paired variables. Dependence is therefore evaluated through concordance of clustering structure rather than covariance, rank order, or shared information.

This formulation is useful when samples are organized into distinct subpopulations. For example, two genes may show little overall correlation across all cells, yet both divide the dataset into the same cellular groups. CCC treats this shared partitioning as evidence of dependence even when a global relationship is weak or absent.

However, clustering-based estimators inherit the sensitivity of clustering algorithms to data representation and parameter choice. Consequently, inferred associations may vary with clustering resolution, distance metric, and local sample structure, making inferred dependence sensitive to clustering specification.

### 2.7. From Nonlinear Correlation to Systems-Level Inference

Modern omics studies increasingly seek to characterize cellular populations, developmental trajectories, regulatory networks, and interactions across multiple molecular layers rather than just individual pairwise associations.

Modern omics workflows increasingly incorporate dependence measures within larger inference systems. In such contexts, measures of association may be used to construct similarity graphs, identify local neighborhoods, quantify cross-modal relationships, or define inputs for downstream inference methods [20,21]. Under these workflows, dependence estimation serves less as a final objective and more as a component of larger inference systems. The focus shifts from individual associations toward reconstruction of higher-order biological organization.

**Figure 1:**
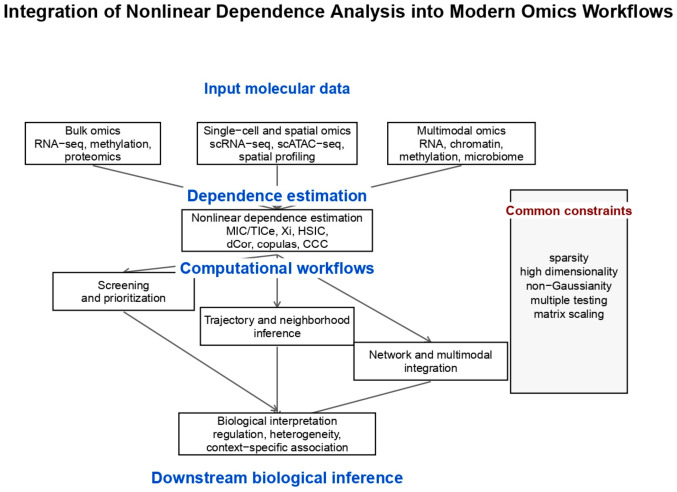
Integration of Nonlinear Dependence Analysis into Modern Omics Workflows. Nonlinear dependence estimation increasingly functions within multimodal, graph-based, and single-cell analytical pipelines rather than as an isolated exploratory procedure. Different methodological classes support distinct forms of transcriptomic, temporal, and systems-level inference across heterogeneous molecular datasets.

Recent developments in graph learning, multimodal integration, and representation learning extend this trend further by modeling biological structure directly within learned feature spaces [43]. Dependence estimation therefore remains important, but increasingly operates within broader systems-level frameworks. Figure 1 summarizes the integration of nonlinear dependence analysis into contemporary omics inference workflows.

Collectively, these developments illustrate the diversity of inferential frameworks now used to characterize biological relationships. Table 1 summarizes their principal trade-offs and provides the foundation for the comparative analysis developed in Section 3.

Major nonlinear dependence frameworks organized according to inferential objective, principal statistical strengths, analytical limitations, and representative omics applications. The table emphasizes how different methodological families optimize distinct inferential priorities rather than a universal performance criterion.

#### Representation Learning and Neural Dependence Estimation

Representation-learning approaches shift the object of inference from explicit dependence estimation to latent representations that encode dependence structure. Instead of evaluating individual associations, they model cellular and molecular organization within latent spaces. Latent spaces are lower-dimensional representations learned from high-dimensional molecular data in which biologically similar cells, genes, or samples occupy nearby positions. In this framework, nonlinear biological relationships are represented geometrically: entities linked through regulatory, developmental, or functional dependencies are embedded close together even when such relationships cannot be captured by simple pairwise association measures.

This trend has been particularly pronounced in single-cell omics. Foundation models such as Geneformer, scGPT, scFoundation, and CellFM are trained on millions of cells to learn general representations of cellular state [44,45,46,47]. These models support cell annotation, perturbation prediction, regulatory-network analysis, and cross-dataset transfer learning.

The conceptual link between nonlinear dependence analysis and modern representation learning lies in a shift in the object of inference. Classical methods quantify dependence directly between observed variables, such as gene-expression levels, protein abundances, or multivariate molecular profiles. Modern foundation models instead infer representations in which dependence structure is encoded implicitly. In this setting, dependence measures are often applied not to raw molecular variables but to learned embeddings. For example, mutual information may be estimated between latent representations, while kernel- and distance-based measures can quantify dependence between high-dimensional feature embeddings generated by neural networks. This transition is particularly evident in information-theoretic approaches, where neural estimators extend mutual-information analysis to high-dimensional representations [48], and in contrastive learning frameworks that use dependence-derived objectives to associate related observations in latent space [49]. Similar principles underlie multimodal integration methods that align transcriptomic, epigenomic, proteomic, or spatial measurements originating from the same biological system [20,22].

Whereas classical nonlinear methods compress dependence into an explicit statistic, modern representation-learning approaches use dependence-derived objectives to shape latent embeddings, allowing information about biological relationships to be distributed across an entire representation rather than summarized by a single coefficient.

Consequently, foundation models can be viewed as a generalization of nonlinear inference: rather than estimating dependence for individual molecular features, they learn representations that organize biological systems according to patterns of statistical dependence.

## 3. Fundamental Challenges in Nonlinear Dependence Inference

**Figure 2:**
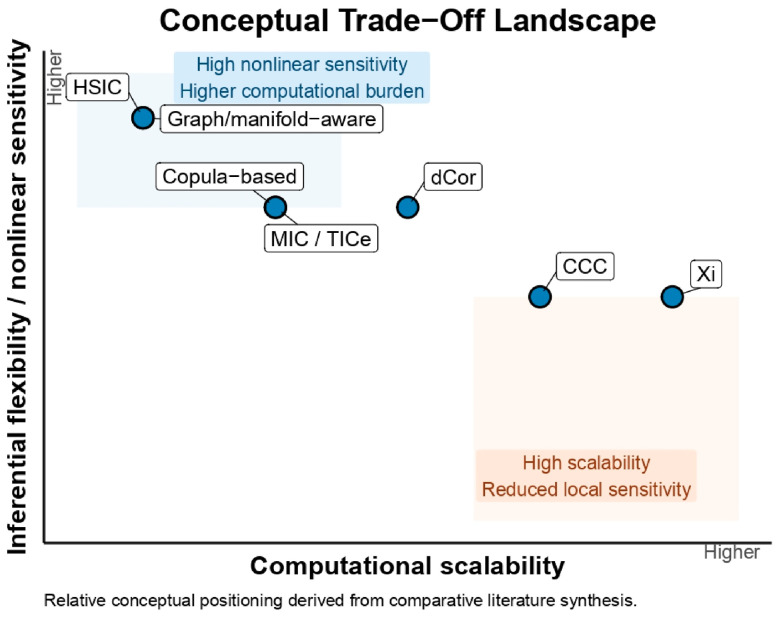
Conceptual Trade-Off Landscape of Nonlinear Dependence Methods. Representative nonlinear dependence frameworks positioned according to approximate computational scalability and nonlinear sensitivity. The figure highlights a common trade-off between sensitivity to complex nonlinear structure and computational efficiency.

Although nonlinear dependence methods differ in their assumptions and objectives, practical performance is often shaped by a smaller set of recurring inferential challenges. Figure 2 illustrates one such trade-off, namely between computational scalability and nonlinear sensitivity across major methodological families. Additional considerations, including equitability, robustness, and heterogeneity, are discussed in the following sections.

### 3.1. Statistical Power, Equitability, and Robustness

Statistical power, equitability, and robustness represent distinct objectives in nonlinear dependence inference. Statistical power concerns the ability to detect departures from independence, equitability concerns the assignment of comparable scores to relationships with similar noise levels despite differences in functional form, and robustness concerns the stability of inferential conclusions under perturbations of the observed data [2,6,15,37]. Because these objectives emphasize different aspects of estimator performance, they frequently generate competing methodological priorities.

The distinction between power and equitability is particularly evident in information-theoretic frameworks. MIC was developed to assign similar scores to relationships with comparable noise levels despite differences in relationship geometry, making it useful for ranking heterogeneous associations [2,15]. However, the adaptive partitioning required for equitability may increase estimator variance under sparse sampling conditions.

Methods optimized for independence testing adopt a different strategy. TICe aggregates information across partition structures to improve power against broad nonlinear alternatives [15]. Similarly, dCor, copula-based estimators, kernel-based approaches such as HSIC, and rank-based methods such as Xi prioritize detection of nonlinear dependence rather than comparability across relationship geometries, although they differ substantially in the assumptions, representations, and statistical mechanisms used to achieve this objective [3,6,17].

Robustness concerns the stability of dependence estimates under perturbations of the observed data, model representation, or estimation procedure. This issue is particularly relevant for modern nonlinear dependence methods because flexibility is frequently achieved through adaptive partitioning, local neighborhoods, similarity structures, latent representations, or learned embeddings. Consequently, the same mechanisms that increase sensitivity to complex dependence patterns may also increase sensitivity to perturbations in the underlying data-generating process [6,37].

Viewed broadly, robustness reflects the extent to which evidence for dependence remains invariant across plausible representations of the same biological system. This perspective extends beyond classical notions of contamination and outlier resistance. Recent developments in robust dependence estimation, representation learning, and systems-level inference increasingly emphasize stability under perturbation as a prerequisite for reproducible inference [21,22,37]. Robustness therefore introduces a fundamental distinction between dependence that is detectable and dependence that remains stable across alternative realizations, representations, and analytical workflows.

### 3.2. High Dimensionality, Sparsity, and Statistical Calibration

High dimensionality alters dependence estimation by shifting the primary constraint from relationship characterization to information availability. As the number of variables increases, the number of candidate associations grows rapidly, while the effective sample size available for estimating each relationship often becomes limiting. Statistical estimation therefore becomes increasingly sensitive to sparsity, noise, and multiple-testing burden. Consequently, high dimensionality influences estimator stability, statistical power, computational feasibility, and inferential calibration across nearly all nonlinear dependence frameworks.

These effects manifest differently across methodological families. Some estimators are limited primarily by sparse sampling and variance inflation, whereas others are affected by distance concentration, similarity-matrix construction, dependence-model specification, or computational scaling.

Information-theoretic methods are particularly sensitive to finite-sample instability because adaptive partition estimation can increase variance under sparse sampling conditions [15]. Kernel-based approaches exhibit related sensitivity governed by bandwidth selection and similarity geometry, which can influence both statistical power and estimator stability [7]. In distance-correlation analysis, distance concentration may reduce testing power as dimensionality increases, although recent theoretical work suggests that power depends jointly on sample size, dependence strength, and statistical calibration rather than on dimensionality alone [36]. Copula-based estimators may likewise become sensitive to dependence-family specification and numerical optimization in high-dimensional settings.

Rank-based approaches partially mitigate some of these challenges by avoiding explicit density estimation and large similarity-matrix construction, thereby improving computational stability. However, this simplification may reduce sensitivity to weak smooth dependence and fine-scale local structure relative to more flexible nonlinear estimators [6]. In this sense, performance in high-dimensional settings is often governed more by information scarcity than by dependence complexity itself.

### 3.3. Computational Scalability and Workflow Integration

Computational scalability concerns the feasibility of dependence estimation as data size, dimensionality, and analytical complexity increase. Computational scalability has become a major determinant of method applicability in modern omics analysis. As transcriptomic, single-cell, and multimodal datasets continue to expand in size, practical performance increasingly depends not only on statistical properties but also on runtime complexity, memory requirements, parallelization strategies, and workflow integration. Consequently, scalability influences which dependence measures remain feasible at genome-wide and systems-level scales.

Scalability differs substantially across methodological families. Information-theoretic approaches often incur substantial optimization costs because dependence estimation requires adaptive partitioning procedures, whereas kernel- and distance-based methods are frequently constrained by similarity-matrix or pairwise-distance computation. Copula-based approaches may likewise become computationally demanding because of dependence-model estimation and numerical optimization requirements [7,13,15]. In contrast, rank-based approaches generally support efficient large-scale screening because dependence is evaluated through ordinal structure rather than explicit reconstruction of relationship geometry [3].

Recent developments increasingly address scalability through computational infrastructure rather than estimator design alone. Parallel computing, approximate inference, distributed workflows, and representation-learning frameworks have enabled nonlinear dependence analysis at scales that would be impractical using standalone estimators. In particular, graph-based and deep-learning approaches shift part of the computational burden from explicit dependence estimation toward learned representations, allowing scalable integration of large multimodal datasets through latent embeddings [44,45,46,47].

From a comparative perspective, scalability introduces a trade-off between statistical flexibility and computational feasibility. Methods that achieve greater adaptivity through partition optimization, complex similarity representations, or learned embeddings frequently incur increased computational cost. Consequently, practical method selection depends not only on statistical performance but also on compatibility with available computational infrastructure and downstream analytical workflows. Table 2 summarizes the principal trade-offs among major nonlinear dependence frameworks. Modern scalability increasingly depends on representation and computational infrastructure rather than estimator design alone.

Comparative inferential trade-offs across major nonlinear dependence frameworks. Methods are contrasted according to nonlinear sensitivity, robustness to noise, computational scalability, interpretability, and characteristic analytical limitations relevant to transcriptomic, multimodal, and single-cell omics analysis.

### 3.4. Heterogeneity and Dependence Structure

Heterogeneity is a pervasive characteristic of modern omics datasets and arises from cellular subpopulations, tissue-specific expression programs, developmental trajectories, disease states, multimodal measurements, and context-specific regulatory processes [21,22]. From a statistical perspective, heterogeneity implies that dependence structure may differ across subsets of observations rather than being represented by a single global relationship. For example, a regulatory association may be strong in one cell type or tissue but weak or absent in another. Consequently, a dependence measure estimated across an entire dataset may reflect an average of multiple distinct association patterns.

This distinction has important implications for nonlinear inference. Many dependence measures implicitly assume that a single relationship generates all observations. Heterogeneity violates this assumption because different subsets of observations may be generated by fundamentally different dependence structures. Consider an extreme example in which a regulator and its target are strongly positively associated in one cell type and strongly negatively associated in another. Within each cell type the relationship is clear, yet when both populations are analyzed together the opposing associations may cancel, producing a near-zero global dependence estimate. Conversely, an apparent dependence detected across a pooled dataset may arise solely because distinct cell types or tissues differ in their average expression levels, even when no meaningful relationship exists within either group. In this sense, heterogeneity changes the meaning of dependence itself: the question is no longer whether two variables are associated, but whether a single dependence structure adequately describes the population being studied.

Methods differ substantially in their response to heterogeneous dependence structures. Information-theoretic, rank-based, distance-based, and copula-based approaches typically summarize dependence across all observations and therefore emphasize population-level structure. In contrast, clustering-based methods, graph-based inference frameworks, and representation-learning approaches may be more sensitive to subgroup-specific organization because they explicitly incorporate local neighborhoods, partitions, or latent structure [12,13,44].

Consequently, heterogeneity introduces a fundamental trade-off between global and local inference that is distinct from the trade-offs between equitability, statistical power, robustness, and computational scalability discussed above. Methods optimized for population-level estimation provide stable summaries of overall association but may obscure subgroup-specific relationships. Methods optimized for local structure may better recover context-dependent dependence patterns but often become more sensitive to sampling variability, parameter selection, and representation choice. The optimal method therefore depends not only on the form of dependence being studied, but also on the level of biological organization at which inference is intended.

Taken together, robustness, dimensionality, scalability, and heterogeneity represent four distinct sources of inferential uncertainty that shape the practical behavior of nonlinear dependence measures. These challenges provide a common framework for interpreting methodological trade-offs and motivate the scenario-based recommendations presented in Section 5.

## 4. Biological Applications and Inferential Perspectives in Molecular Sciences

The following examples illustrate how nonlinear dependence methods address major biological and inferential challenges in modern molecular datasets. Rather than emphasizing individual estimators, the examples highlight how different methodological families provide complementary perspectives on common biological problems.

### 4.1. Nonlinear Regulatory and Epigenetic Relationships

Okada et al. applied the Maximal Information Coefficient (MIC) within the DICNAP workflow to identify nonlinear age-associated DNA methylation trajectories in human datasets [50]. The analysis identified progressive demethylation patterns that decelerated after approximately 60 years of age and revealed increased methylation variability during aging. These results demonstrated sensitivity of MIC-based inference to nonlinear methylation dynamics not detected by linear correlation analysis.

Additional applications of information-theoretic nonlinear dependence methods in transcriptomic and molecular studies are summarized in Supplementary Table S3.

### 4.2. Temporal and Oscillatory Biological Processes

Pividori et al. applied Chatterjee’s Xi to yeast cell-cycle transcriptomic data to identify oscillatory transcriptional dynamics across 23 consecutive time points [13]. Compared with MIC, distance correlation, HHG, and HSIC, Xi showed improved sensitivity for cyclic expression patterns and identified additional oscillatory genes not prioritized by competing methods. These results illustrate the utility of rank-based inference for transcriptomic screening. Additional applications of rank-based nonlinear dependence analysis, including Chatterjee’s Xi in molecular and single-cell studies, are summarized in Supplementary Table S4.

### 4.3. Heterogeneous Cellular Populations and Context-Dependent Structure

Heterogeneous cellular populations provide a practical example of the inferential challenges discussed in Section 3.4. In single-cell transcriptomic datasets, dependence structures may differ across cellular states, developmental trajectories, or tissue contexts, making global association measures difficult to interpret.

Kernel-based approaches are increasingly integrated with neighborhood graphs and trajectory analysis to identify context-dependent regulatory interactions across differentiating cellular populations [7]. By evaluating relationships within local transcriptomic neighborhoods, these methods improve sensitivity to local transcriptomic structure associated with cellular transitions and developmental trajectories. However, performance remains sensitive to kernel specification, neighborhood definition, and computational scaling in large datasets.

A complementary strategy focuses on subgroup organization rather than local similarity. Pividori et al. introduced the Clustermatch Correlation Coefficient (CCC), which estimates dependence through concordance of clustering structure rather than covariance-based association [13]. In GTEx whole-blood RNA-seq data, CCC identified subgroup-specific co-expression patterns that were not detected by Pearson correlation, Spearman correlation, or MIC-based analysis. Many of these relationships replicated in independent tissue-specific networks, supporting the utility of clustering-based inference in heterogeneous transcriptomic datasets.

Together, these examples illustrate complementary approaches to context-dependent inference. Kernel-based methods recover local dependence through neighborhood structure, whereas clustering-based methods identify dependence through shared subgroup organization. Both approaches can reveal biologically meaningful relationships that may be obscured by population-level summaries and are therefore particularly valuable when dependence varies across cellular states, trajectories, or biological subpopulations.

### 4.4. Multimodal Molecular Integration

Multimodal molecular integration increasingly requires methods capable of identifying associations across heterogeneous molecular measurements, including transcriptomic, epigenomic, proteomic, microbiome, and chromatin-accessibility data. Distance-based approaches such as dCor have been applied to transcriptomic feature screening and multimodal omics integration because they detect nonlinear dependence without requiring specification of functional form [11]. These methods are particularly useful for identifying multivariate associations distributed across weak nonlinear effects rather than dominant monotonic trends. However, testing power may decline because of distance concentration effects in high-dimensional settings (Section 3.2).

Copula-based approaches provide an alternative inferential strategy for the same biological problem because they model dependence structure independently of marginal distributions [12,42]. Applications include integration of RNA-seq, chromatin accessibility, DNA methylation, and microbiome profiles under mixed distributional structure. Unlike distance-based approaches, which characterize dependence through pairwise distance relationships, copula-based methods explicitly separate dependence structure from marginal behavior, allowing integration of heterogeneous molecular measurements with non-Gaussian marginals and mixed variable types. However, these advantages may come at the cost of increased computational complexity in large-scale settings (Section 3.3).

Taken together, distance-based and copula-based approaches illustrate complementary strategies for multimodal integration: distance-based methods emphasize flexible detection of general nonlinear associations, whereas copula-based methods emphasize explicit modeling of dependence across heterogeneous data distributions.

### 4.5. Systems-Level and Network Inference

Gene regulatory networks and multimodal molecular interaction networks provide a natural setting for nonlinear dependence analysis because biological regulation is often distributed across many interacting components rather than isolated gene pairs. Information-theoretic methods have been widely applied to network reconstruction because they detect nonlinear associations without requiring specification of functional form [2,15,24]. Copula-based approaches have similarly been used for transcriptomic and multimodal network inference, particularly when dependence must be modeled across heterogeneous molecular measurements with different marginal distributions [12,42].

Recent studies increasingly integrate dependence estimation with graph-based network construction and multimodal inference workflows [21,27,28]. Representation-learning frameworks further extend these applications by supporting systems-level analysis across large cellular and molecular datasets [44,45,46,47]. Collectively, these applications illustrate how nonlinear dependence analysis contributes to reconstruction of regulatory and multimodal biological networks.

### 4.6. Lessons from Comparative Benchmarking and Workflow Evaluation

Across these application domains, comparative benchmarking provides insight not only into estimator performance but also into the broader inferential trade-offs discussed in Section 3. The benchmarking studies compiled in Supplementary Table S2 and the real-world comparative evaluations summarized in Supplementary Table S5 provide a unique perspective on nonlinear dependence analysis because they evaluate competing frameworks under common biological and statistical settings. Although individual studies were designed to assess specific methods, collectively they reveal broader principles governing method performance.

Analyses of transcriptomic and systems-biology datasets show that different methods recover different aspects of biological organization, with substantial variation in sensitivity to nonlinear, heterogeneous, and context-dependent relationships [13]. Rather than converging on a single optimal estimator, real-data studies demonstrate that biological structure can be represented through multiple inferential perspectives.

The simulation studies summarized in Supplementary Table S2 reinforce this conclusion. Comparative evaluations of MIC, TICe, mutual information, distance correlation, HSIC, HHG, Xi, and related approaches consistently show that no nonlinear dependence measure performs optimally across all relationship geometries, noise regimes, dimensionalities, and sample sizes [3,4,6]. Instead, benchmarking reveals recurring trade-offs among flexibility, robustness, computational efficiency, and sensitivity to biological structure, closely mirroring the theoretical considerations discussed in Section 3.

Collectively, the studies summarized in Supplementary Table S2 suggest that method selection should be guided by analytical objectives and data characteristics rather than universal performance rankings. More recent benchmarking efforts further indicate a shift from evaluating individual dependence estimators toward assessing complete inference workflows involving graph learning, multimodal integration, and representation learning [21,27,28,43]. As a result, benchmarking is increasingly focused on the biological utility of learned representations rather than the performance of individual association statistics.

## 5. Practical Decision Framework for Method Selection

Method selection in nonlinear dependence analysis is best viewed as a decision problem involving inferential objectives and analytical constraints rather than as a purely methodological choice among competing families of estimators. Because different methods prioritize distinct objectives—including dependence detection, equitable characterization, robustness, and scalability—and respond differently to dimensionality and heterogeneity, the optimal framework depends on the primary scientific objective and the dominant source of inferential uncertainty. Table 3 summarizes this decision framework.

Objective-oriented decision matrix linking inferential goals to dominant analytical constraints, recommended methodological families, and principal trade-offs. The framework emphasizes that method selection should begin with the scientific objective and source of inferential uncertainty rather than with individual estimators.

### 5.1. Objective: Detect Arbitrary Nonlinear Relationships

Feature selection imposes constraints distinct from exploratory dependence analysis because the primary objective is stable prioritization of predictive variables. In settings characterized by many variables and limited samples (Section 3.2), this increases the risk of unstable ranking and overfitting.

A common strategy combines TICe-based filtering with MICe-based ranking to balance sensitivity to nonlinear dependence with interpretable prioritization across heterogeneous relationship structures [15]. Multivariate MIC extensions may additionally improve detection of synergistic predictor interactions that are weak in marginal analysis [26].

### 5.2. Objective: Stable Inference Under Limited Data

Small-sample omics studies are vulnerable to estimator instability, finite-sample bias, and overfitting. Under these conditions, inferential stability often becomes more important than maximal sensitivity.

Rank-based approaches such as Chatterjee’s Xi are useful because they avoid explicit density estimation and remain computationally tractable under limited sample size [3]. TICe may additionally be useful when the primary objective is independence testing rather than comparative ranking [15]. By contrast, MIC-derived, kernel-based, and copula-based methods require greater caution because partition optimization, bandwidth specification, and dependence-model estimation become increasingly unstable with limited sampling [7,12].

### 5.3. Objective: Large-Scale Screening

In large-scale omics studies, the primary challenge is often not sensitivity to complex dependence structure but computational scalability. Transcriptomic, single-cell, and multimodal datasets routinely require evaluation of many candidate associations, making runtime complexity, memory requirements, and workflow integration major determinants of method feasibility.

Under these conditions, methods that support efficient large-scale screening are generally preferred for initial analysis. Rank-based statistics and screening-oriented information-theoretic methods are particularly useful because they avoid large similarity matrices while supporting efficient computation [3,15]. Approximate MIC variants and related low-complexity estimators further reduce computational burden in large datasets [30,31].

More computationally intensive methods are often applied after candidate reduction. Distance correlation remains useful because it detects nonlinear multivariate dependence without requiring specification of functional form [51]. Kernel-based methods are generally more practical after candidate reduction or dimensionality compression because kernel operations scale with sample number and neighborhood density [7]. Similarly, graph-based and representation-learning frameworks increasingly support atlas-scale analysis by shifting computation toward learned representations and scalable integration workflows [52].

Consequently, method selection in large-scale screening is often determined less by theoretical flexibility than by computational feasibility. Practical workflows therefore frequently combine computationally efficient nonlinear screening with graph-aware or biologically constrained downstream analysis.

### 5.4. Objective: Integrate Multiple Data Modalities

Integrative omics studies increasingly combine transcriptomic, epigenomic, microbiome, imaging, clinical, and phenotypic variables across mixed data types.

Clustering-based approaches such as CCC are useful when dependence is expressed through subgroup structure rather than smooth functional association [13]. Copula-based methods provide another option because they model dependence separately from marginal distributions and can accommodate mixed discrete-continuous molecular data [12,42]. Rank-based approaches such as Xi may additionally be advantageous when transformation invariance and distributional robustness are desirable [3].

By contrast, information-theoretic and kernel-based methods become increasingly sensitive to sparse category combinations, encoding strategy, and hyperparameter specification in mixed-data settings [7].

### 5.5. Objective: Systems-Level and Network Inference

Gene regulatory and co-expression network inference requires methods that remain stable across many weak associations. Information-theoretic statistics, dCor, and copula-based estimators are useful when regulatory relationships are nonlinear, non-Gaussian, or distributed across multiple molecular layers [12,15,42].

Kernel and graph-based methods may improve recovery of local or higher-order network structure, but usually require candidate reduction, sparsification, or regularization before application at large analytical scale [27,28]. In practice, nonlinear dependence measures are most useful as network-filtering or edge-prioritization steps followed by stability selection, biological constraint, or independent validation.

### 5.6. Objective: Recover Local or Context-Dependent Structure

Single-cell and spatial omics introduce sparsity, compositional effects, spatial neighborhoods, and trajectory structure.

Rank-based methods provide scalable screening under sparse expression, whereas kernel and graph-based approaches are useful when inference depends on neighborhood structure, pseudotime, or spatial proximity [53,54,55]. Copula-based methods are useful for single-cell multi-omics integration because they model dependence separately from marginal distributions and can accommodate mixed discrete-continuous measurements [12]. dCor provides a complementary option for multimodal association testing when nonlinear dependence is expected but functional form is unknown.

An extended practical decision framework incorporating additional analytical scenarios, inferential limitations, and implementation considerations is provided in Supplementary Table S7. Additional representative single-cell and spatial omics applications of nonlinear dependence analysis are summarized in Supplementary Table S8.

### 5.7. Objective: Time-Series and Oscillatory Data

Time-resolved omics datasets often contain phase shifts, transient activation, and nonlinear temporal trajectories that are not adequately represented by linear correlation.

When oscillatory behavior and approximate waveform structure are expected, template-aware approaches such as MICOP improve sensitivity to rhythmic patterns under sparse and noisy sampling conditions [56]. Exploratory analyses with uncertain temporal structure instead benefit from model-agnostic approaches such as Chatterjee’s Xi, which detects nonlinear temporal dependence without requiring explicit assumptions regarding periodicity or waveform geometry [3].

Kernel and graph-aware methods further support trajectory-based analysis in single-cell workflows by integrating dependence estimation with pseudotime and neighborhood representations [53,54]. Additional representative methods for oscillatory and temporal dependence analysis are summarized in Supplementary Table S6.

No nonlinear dependence framework is universally optimal because different methods address different sources of inferential uncertainty. Method selection should therefore begin with the analytical objective and the dominant constraints imposed by robustness, dimensionality, scalability, and heterogeneity rather than with the choice of a particular estimator. Viewed in this way, nonlinear dependence measures function as components of broader inferential workflows rather than isolated statistical tools.

## 6. Software Ecosystem and Infrastructure Considerations

The practical use of nonlinear dependence measures in molecular sciences depends not only on statistical performance, but also on computational feasibility, reproducibility, and scalability. In transcriptomic, single-cell, and multimodal datasets, algorithmic complexity, memory usage, numerical stability, and support for parallel computation directly influence which forms of nonlinear dependence can be analyzed at scale.

### 6.1. Statistical and Exploratory Analysis Environments

The R ecosystem remains central for statistically oriented nonlinear inference because of its integration with Bioconductor workflows and transcriptomic analysis pipelines. The package *minerva* implements MIC, MICe, and TICe together with permutation-based significance testing [2,15], whereas *XICOR* provides efficient implementations of Chatterjee’s Xi [3]. Distance correlation methods are available through packages such as *energy* [39], whereas Gaussian copula workflows are supported through *copula* [57], *huge* [58], and related frameworks for mixed-type omics data.

Rank-based approaches generally scale more efficiently than adaptive information-theoretic, kernel-based, and copula-based estimators because they avoid density estimation and large optimization procedures.

### 6.2. Scalable and Machine-Learning-Oriented Workflows

As omics datasets increased in scale, nonlinear inference increasingly relied on infrastructures optimized for sparse computation, distributed execution, and machine-learning interoperability. Python-based environments became important because they integrate naturally with graph learning, GPU-aware computation, and large-scale single-cell analysis.

Parallelized implementations of MICe and TICe support high-throughput nonlinear screening, whereas clustering-based approaches such as CCC provide optimized workflows for large transcriptomic datasets [13]. Kernel-, distance-, and copula-based methods additionally integrate efficiently with numerical and machine-learning libraries for graph inference, multimodal integration, and large-scale association analysis.

Additional software ecosystems, workflow-management environments, and computational infrastructures used in nonlinear dependence analysis are summarized in Supplementary Table S9.

### 6.3. Reproducibility and Cross-Platform Consistency

Many nonlinear estimators are sensitive to partition initialization, floating-point precision, randomized approximation procedures, kernel parameterization, and execution order [2]. Reproducible analyses therefore require explicit reporting of software versions, preprocessing procedures, estimator settings, random seeds, and permutation strategies.

Large-scale transcriptomic and single-cell workflows commonly rely on containerized and workflow-managed environments such as Docker, Singularity/Apptainer, Nextflow, and Snakemake to standardize nonlinear inference across heterogeneous hardware systems [59,60].

### 6.4. Memory Usage and Resource-Aware Inference

Resource-efficient computation has become a major practical constraint in nonlinear omics analysis, particularly for methods requiring large similarity, distance, kernel, or partition matrices. Recent implementations therefore use sparse matrix representations, approximate kernels, blockwise estimation, streaming computation, low-rank decompositions, and single-instruction multiple-data (SIMD) vectorization to improve scalability while limiting memory usage.

Information-theoretic workflows based on MICe and TICe benefit from embarrassingly parallel execution across variable pairs, whereas rank-based statistics such as Chatterjee’s Xi remain comparatively lightweight because they avoid density estimation and large matrix operations [3]. By contrast, kernel-, distance-, and copula-based approaches may require substantially greater computational resources because of matrix scaling, iterative optimization, and covariance regularization in high-dimensional settings [7,52].

Large-scale omics workflows commonly apply feature filtering and dimensionality reduction before nonlinear dependence analysis because many methods scale poorly in large datasets. Memory usage, optimization complexity, and computational scalability therefore strongly influence practical method selection in transcriptomic, multimodal, and single-cell studies, as summarized in Table 4.

Representative computational characteristics affecting scalability, reproducibility, implementation feasibility, and software accessibility of nonlinear dependence workflows in modern omics analysis. The table summarizes major computational bottlenecks, optimization strategies, and representative software ecosystems across methodological families commonly used in transcriptomic, multimodal, and single-cell inference.

### 6.5. Limitations and Future Directions

Despite major advances in nonlinear dependence analysis, no existing framework fully resolves the combined statistical, computational, and interpretability challenges posed by modern omics data. Nonlinear inference therefore remains governed by trade-offs among sensitivity, robustness, scalability, and biological interpretability.

As summarized in Supplementary Table S1, current methodological development is concentrated primarily in kernel-based dependence frameworks, modern distance-correlation methods, and emerging clustering-based and subgroup-aware approaches, whereas measures such as MIC increasingly serve as mature benchmark methodologies.

### 6.6. Statistical Calibration and Multiple Testing

High-dimensional omics datasets substantially increase the risk of spurious nonlinear associations because the number of candidate relationships grows combinatorially with dimensionality. Adaptive partitioning, kernel-based, and distance-based estimators are especially sensitive because estimation error accumulates across large comparison spaces [2,15,35].

Increasing dimensionality additionally reduces estimator stability and inflates sample-size requirements, particularly for entropy- and kernel-based approaches sensitive to sparse sampling geometry [7]. Pairwise dependence analysis may also miss higher-order interactions while inflating false discovery rates in large-scale screening studies.

Permutation-based inference and FDR correction remain widely used but become computationally burdensome in large datasets and may yield unstable calibration under limited sampling. Progress will require multivariate and structured inference frameworks integrating dimensionality reduction, sparsity-aware regularization, and computationally efficient statistical calibration.

### 6.7. Interpretability and Inferential Stability

A recurring challenge in nonlinear dependence analysis is that increased representational flexibility often comes at the cost of interpretability and inferential stability. Similar dependence scores may arise from fundamentally different biological relationships, while methods capable of detecting nonlinear structure may also become more sensitive to noise and sampling variability [7,12,15]. Consequently, robust analysis requires evaluation of reproducibility alongside detection performance through cross-validation, stability selection, permutation testing, and regularization.

### 6.8. Dynamic and Single-Cell Inference

Dynamic and single-cell omics data expose limitations in dependence measures originally developed for static bulk molecular profiling. Biological rhythms are frequently irregular, non-stationary, sparsely sampled, and variable across cells or conditions, complicating robust inference of temporal dependence.

Template-based approaches such as MICOP improve sensitivity to rhythmic structure by comparing observed trajectories against predefined oscillatory waveforms [56]. More flexible rank-, kernel-, distance-, and copula-based methods avoid explicit waveform assumptions and can detect broader temporal and multimodal relationships, although they often reduce interpretability or increase computational cost.

Single-cell datasets introduce additional challenges including dropout noise, sparse count structure, compositional heterogeneity, and uncertain pseudotemporal ordering. Recent methods increasingly integrate nonlinear dependence estimation with graph learning, neighborhood-aware modeling, and multimodal integration to characterize regulatory relationships across heterogeneous cellular states [53,54].

Scalable integration of dynamic, multimodal, and spatially structured molecular data remains an important unresolved problem.

## 7. Conclusions

Nonlinear dependence methods have become important tools for modern omics analysis, but no single framework is optimal across all biological settings. Rather than reflecting differences in estimator design alone, practical performance is shaped by recurring inferential challenges including dimensionality, scalability, robustness, and biological heterogeneity. Consequently, method selection should be guided by the scientific objective, data characteristics, and the dominant sources of inferential uncertainty.

Recent developments in multimodal integration, graph-based learning, and foundation models further suggest a shift from isolated pairwise associations toward systems-level representations of biological dependence. Future progress will depend not only on methodological innovation but also on improved statistical calibration, reproducibility, scalability, and integration with emerging data modalities.

## Figures and Tables

**Table 1:** Inferential Characteristics of Major Nonlinear Dependence Frameworks.

Method Class	Representative Methods	Inferential Objective	Key Strengths	Main Limitations	Representative Omics Applications
Information-theoretic	MIC, MICe, TICe	Model-free nonlinear dependence estimation	Sensitive to heterogeneous functional relationships	Partition instability; sample-size sensitivity	DNA methylation, co-expression screening
Rank-based	Xi, ordinal nonlinear statistics	Robust ordinal dependence estimation	Computational efficiency; robustness to outliers	Reduced sensitivity to weak smooth structure	Transcriptomic screening, oscillatory expression analysis
Kernel-based	HSIC, RKHS-based methods	Local and high-order dependence estimation	Captures complex local structure	Kernel specification and matrix scaling	Single-cell trajectory analysis
Distance-based	dCor, energy statistics	General dependence estimation via distance structure	Distribution-free nonlinear dependence estimation	Quadratic scaling in large datasets	Multimodal omics integration
Copula-based	Gaussian copula, semiparametric copulas	Dependence modeling independent of marginals	Handles mixed and non-Gaussian distributions	High-dimensional parameter estimation	Single-cell multi-omics integration
Clustering-based	CCC, subgroup-aware methods	Subgroup-specific dependence estimation	Captures multimodal co-expression structure	Dependence on clustering quality	tissue-specific networks
Graph-/network-aware	Graph learning, manifold-based inference	Context-aware systems-level inference	Integrates neighborhood and multimodal structure	Reduced interpretability; high computational cost	Single-cell and spatial omics
Representation-learning	Geneformer, scGPT, scFoundation, CellFM	Learning biologically informative latent representations	Captures complex regulatory, cellular, and multimodal structure; supports transfer learning	Reduced interpretability; high computational requirements; large training datasets	Cell-state annotation, perturbation prediction, multimodal integration, foundation-model inference

**Table 2:** Statistical Trade-Offs Among Nonlinear Dependence Methods.

Method Class	Nonlinear Sensitivity	Robustness to Noise	Scalability	Interpretability	Common Inferential Limitation
Information-theoretic	High	Moderate	Moderate to low	Moderate	Estimator instability under sparse sampling
Rank-based	Moderate	High	High	High	Limited sensitivity to weak smooth dependence
Kernel-based	High	Moderate	Low to moderate	Low	Sensitivity to kernel parameterization
Distance-based	High	Moderate	Moderate	Moderate	Matrix scaling and computational burden
Copula-based	High	Moderate	Low	Moderate	Computational complexity in high dimensions
Clustering-based	Moderate to high	Moderate	Moderate	Moderate	Sensitivity to clustering structure
Graph-/network-aware	High	Moderate	Low	Low	Reduced mechanistic transparency

**Table 3:** Decision Framework for Selecting Nonlinear Dependence Methods in Omics Studies.

Inferential Objective	Dominant Constraint	Recommended Method Family	Principal Trade-Off
Detect arbitrary nonlinear relationships	Relationship-geometry uncertainty	Information-theoretic (MI, MICe, TICe); distance-based methods	Greater estimator instability and computational cost
Stable inference under limited data	Robustness and finite-sample stability	Rank-based methods (Xi); conservative information-theoretic testing	Reduced sensitivity to weak smooth dependence and fine-scale structure
Large-scale screening	Computational scalability	Rank-based methods; screening-oriented information-theoretic methods (TICe)	Initial filtering may sacrifice local or higher-order structure
Recover local or context-dependent structure	Heterogeneity and subgroup organization	Clustering-based, kernel-based, and graph-aware methods	Sensitivity to parameter choice, representation, and sampling variability
Integrate multiple molecular modalities	Mixed distributions and heterogeneous measurement types	Copula-based, distance-based, and representation-learning approaches	Increased modeling complexity and reduced direct interpretability
Systems-level network inference	Higher-order biological organization and workflow integration	Graph-aware, kernel-based, information-theoretic, and copula-based approaches	Greater computational burden and reduced mechanistic transparency
Atlas-scale multimodal inference	Scalability and dimensionality	Graph-aware and representation-learning approaches	Dependence structure becomes implicit within latent representations
Temporal and oscillatory dependence analysis	Dynamic and nonstationary structure	Rank-based, kernel-based, and specialized temporal methods	Trade-off between model flexibility and temporal interpretability

**Table 4:** Computational and Infrastructure Constraints in Nonlinear Omics Analysis.

Method Class	Primary Computational Burden	Common Optimization Strategies	Representative Software Ecosystems	Scalability	Main Scalability Constraint
Information-theoretic	Adaptive partition estimation and permutation testing	Parallelization; staged screening; heuristic partition search	minerva (R), MICtools (Python)	Moderate	Partition search complexity and repeated resampling
Rank-based	Sorting and rank estimation across large feature spaces	Vectorization; parallel execution; efficient ranking algorithms	XICOR (R)	High	Large pairwise comparison space in high-dimensional transcriptomics
Kernel-based	Kernel matrix construction and optimization	Approximate kernels; sparse representations; low-rank approximation	kernlab (R), scikit-learn (Python)	Moderate	Quadratic matrix scaling and kernel parameter tuning
Distance-based	Distance matrix estimation and storage	Blockwise estimation; low-rank approximation; distributed computation	energy (R)	Moderate	Matrix scaling and memory usage in large multimodal datasets
Copula-based	Covariance regularization and high-dimensional optimization	Sparse estimation; semiparametric approximation; regularization	copula (R), huge (R)	Moderate	High-dimensional parameter estimation and optimization instability
Clustering-based	Repeated clustering and subgroup estimation	Approximate clustering; accelerated nearest-neighbor search	CCC implementations (Python; Numba-accelerated)	High	Dependence on clustering stability and subgroup resolution
Graph-/network-aware	Graph learning, neighborhood inference, and multimodal integration	GPU acceleration; distributed computing; sparse graph representations	Scanpy (Python), PyTorch Geometric (Python)	Variable	Graph construction and multimodal scaling in large single-cell datasets

## Data Availability

No new data were created or analyzed in this study. Data sharing is not applicable to this article.

## Supplementary Materials

The following supporting information can be downloaded at: https://www.mdpi.com/article/10.3390/ijms27135700/s1. References [61,62,63,64,65,66,67,68,69,70,71,72,73,74,75] are cited in the Supplementary Materials.

## Abbreviations

CCC	Clustermatch Correlation Coefficient
DICNAP	Data-Driven Identification and Classification of Nonlinear Aging Patterns
dCor	Distance Correlation
FDR	False Discovery Rate
GTEx	Genotype-Tissue Expression Project
HHG	Heller–Heller–Gorfine test
HSIC	Hilbert–Schmidt Independence Criterion
Kc	Kernelized Correlation
MI	Mutual Information
MIC	Maximal Information Coefficient
MICe	Maximal Information Coefficient estimator
MICOP	Maximal Information Coefficient-based Oscillation Prediction
RBF	Radial Basis Function
RKHS	Reproducing Kernel Hilbert Space
RNA-seq	RNA sequencing
SIMD	Single Instruction, Multiple Data
TIC	Total Information Coefficient
TICe	Total Information Coefficient estimator
UIC	Uniform Information Coefficient
Xi	Chatterjee’s correlation coefficient
