# Supplementary material for: Statistical Methods for Detecting Nonlinear Relationships in Gene Expression and Omics Data: A Review
Source: Int J Mol Sci. 2026 Jun 24;27(13):5700. doi: 10.3390/ijms27135700 (PMC13361526; doi:10.3390/ijms27135700)
Supplement: Supplementary file 1 [file ijms-27-05700-s001.zip › ijms-4241681-supplementary.pdf]

# 1 Supplementary Tables

**Supplementary Table S1. Evolution and Current Status of Major Nonlinear Dependence Measures.**

Representative nonlinear dependence frameworks relevant to molecular and omics research, from foundational developments to current research directions.

| Method / framework | Key publication | Primary contribution | Conceptual significance | Maturity | OMICS adoption | Current research activity | Major current developments |
|---|---|---|---|---|---|---|---|
| Mutual information | Shannon 1948 | Information-theoretic dependence quantification | Foundation for model-free nonlinear association analysis | Established | High | High | Neural MI estimation, conditional MI, high-dimensional estimation |
| Distance correlation | Székely et al. 2007 | General dependence estimation via distance covariance | Distribution-free nonlinear association detection | Established | Moderate | High | Conditional dCor, multivariate dCor, energy statistics |
| Maximal Information Coefficient (MIC) | Reshef et al. 2011 | Equitable nonlinear dependence estimation | Model-free comparison across functional forms | Mature | Moderate | Low | Benchmarking, scalable implementations |
| MICe and TICe | Reshef et al. 2016 | Improved power and statistical calibration | Scalable nonlinear screening and independence testing | Mature | Moderate | Moderate-low | High-dimensional screening, statistical calibration, computationally efficient variants |
| HSIC / RKHS-based inference | Gretton et al. 2005 | Kernel-based dependence estimation | Local and high-order nonlinear structure detection | Active | Growing | Very high | Conditional HSIC, HSIC-Lasso, high-dimensional testing |
| Chatterjee's Xi | Chatterjee 2021 | Rank-based nonlinear dependence statistic | Scalable and robust ordinal dependence inference | Emerging | Growing | Moderate-high | Asymptotic theory, power analysis, extensions |
| CCC | Pividori et al. 2024 | Clustering-aware association estimation | Subgroup-specific and multimodal co-expression detection | Emerging | Limited | Moderate | Heterogeneity-aware dependence |
| Copula frameworks | Ma, Davis and Ho 2023 | Dependence modeling independent of marginals | Mixed-distribution and multimodal omics integration | Active | Growing | Moderate | Sparse and high-dimensional copulas |
| Foundation models / representation learning | [45, 46] | Learning general biological representations from large-scale omics data | Extends nonlinear dependence concepts into latent-space inference and transfer learning | Emerging | Growing rapidly | Very high | Foundation models (Geneformer, scGPT, scFoundation, CellFM), multimodal learning, perturbation prediction, cross-dataset transfer learning |

**Supplementary Table S2. Extended Comparative Benchmarking Studies of Nonlinear Dependence Methods.**

Representative benchmarking and comparative evaluation studies examining statistical performance, equitability, robustness, computational scalability, and inferential behavior of nonlinear dependence measures under heterogeneous simulation settings relevant to transcriptomic, multimodal, oscillatory, and systems-level omics analysis. The table summarizes comparative evaluations of information-theoretic, rank-based, kernel-based, clustering-based, and distance-based frameworks using simulated noisy functional relationships, temporal oscillations, heterogeneous dependence structures, and multidimensional association patterns. Emphasis is placed on the trade-offs among statistical power, computational efficiency, robustness to noise, sensitivity to complex nonlinear structure, and scalability in high-dimensional biological data analysis.

| Indexes benchmarked | Simulated variables used in the benchmark analysis | Conclusions | Reference |
|---|---|---|---|
| MIC, MIC*, MICe, TIC, TICe. | (1) Noisy Functional Relationships: a set of noisy functional relationships was simulated to evaluate the equitability and performance of the proposed coefficients. These relationships encompassed a range of functional forms, including linear, exponential, and periodic functions, with noise added to replicate the complexities and variability typical of real-world data.<br>(2) Gaussian Process Distribution: to assess equitability more objectively, the authors simulated 160 random functions sampled from a Gaussian process distribution using a radial basis function kernel. This approach provided a robust framework for evaluating the performance of the coefficients under a range of different functional forms. | (1) Performance: MICe, an estimator of MIC, demonstrated superior equitability relative to $R^2$ across a broad set of noisy functional relationships. It outperformed existing methods in terms of equitability, both on a manually selected set of noisy functional relationships and on a randomly chosen set, underscoring its robustness and reliability in diverse scenarios.<br>(2) Robustness: MICe was shown to be minimally sensitive to the bandwidth of the Gaussian process from which relationships were drawn. This characteristic contributes to its robustness across a wide range of relationship complexities, enhancing its utility in real-world applications.<br>(3) Bias and variance properties: MICe exhibited superior bias and variance properties when compared to the heuristic algorithm used for calculating the original MIC statistic. Specifically, MICe demonstrated lower expected squared error across a range of $R^2$ values, indicating more consistent and reliable performance across different data types.<br>(4) Performance of TICe: TICe exhibited exceptional power in independence testing, performing at least comparably to, if not better than, existing methods across an index suite of relationships. This underscores TICe's strength in identifying true relationships within complex datasets.<br>(5) Comparison with distance correlation: TICe outperformed distance correlation in 5 out of the 8 relationship types examined, and performed comparably on the remaining three, highlighting its broad applicability and superior performance in detecting associations.<br>(6) MICe vs. TICe: TICe also outperformed both the original MIC and MICe in terms of power against independence, validating the hypothesis that summing all entries in the characteristic matrix (as TICe does) leads to substantial improvements in power over the method of taking the maximum entry (as MICe does). This indicates the potential for TICe to uncover more nuanced associations.<br>(7) Computational efficiency: the algorithms for calculating MICe and TICe were demonstrated to be highly efficient in practice, facilitating the analysis of extremely large datasets. In particular, the EquicharClump | [15] |

| | | | |
|---|---|---|---|
| | | algorithm offered significant speed improvements over the heuristic Approx-MIC algorithm used in earlier MIC computations.<br>(8) Proposed data analysis strategy: the paper proposed a novel data analysis strategy that leverages TICe to filter out non-significant relationships, followed by ranking the remaining relationships using MICe. This two-step approach takes advantage of TICe's powerful testing capabilities to reduce the burden of multiple testing on MICe, while utilizing MICe's superior equitability to rank relationships effectively for further exploration. This combined approach enhances both the efficiency and accuracy of the analysis process. | |
| MICOP, JTK, ARS, LS, and COSOPT. | Simulated time-series data: the performance of MICOP was evaluated on a range of challenging simulated datasets, including:<br>a) Decaying oscillation data: simulated time-series data designed to mimic molecular oscillation decay, where the peak value in the second cycle is one-third of the peak value in the first cycle. This test assessed MICOP's ability to detect oscillations in the presence of amplitude decay.<br>b) Noisy data: time-series data with added noise following a normal distribution (mean = 0, standard deviation = 0.6). This scenario tested MICOP's robustness and sensitivity to high levels of noise.<br>c) Low sampling frequency data: simulated data with varying sampling intervals to evaluate MICOP's robustness and performance under low sampling frequency conditions, which are often encountered in real-world experimental data.<br>d) One-cycle data: simulated datasets containing only a single cycle of oscillation, used to assess MICOP's ability to detect oscillations with limited data points, a common challenge in time-series analysis.<br>These tests provided a comprehensive evaluation of MICOP's performance in detecting oscillatory patterns across different types of data distortions. | MICOP and JTK both exhibit strong performance when applied to one-cycle data, with MICOP frequently outperforming JTK, particularly under challenging conditions such as low sampling frequency and high levels of noise. | [56] |
| MIC, MICe, TICe, $S^{DDP}$, Kraskov | Twelve various types of noise distributions: X noise only, Y noise only, or noise in both variables, as well | The simulation results demonstrated that MICe consistently exhibited high equitability across a range of noise and sample models, as well as varying sample sizes, outperforming other methods in most scenarios. | [61] |

| | | | |
|---|---|---|---|
| Mutual Information Estimator, dCor, Hilbert-Schmidt Information Criterion, $R^2$, maximal correlation, Randomized Dependence Coefficient, HHG. | as various functions including linear, exponential, parabolic, and other types of relationships with varying levels of additive Gaussian noise. These simulated random variables were used to create a diverse set of noisy functional relationships. | TICe and SDDP exhibited state-of-the-art performance in terms of power to detect independence, with several other methods, such as distance correlation, also performing commendably. The analyses revealed a trade-off between power against independence and equitability, suggesting that no single method excels in both dimensions simultaneously. Based on these findings, the study recommended a combined approach: initially using TICe for filtering due to its superior power, followed by ranking the filtered relationships with MICe, which excels in equitability. This integrated strategy offers a balanced and effective framework for exploratory data analysis. | |
| Xi, MIC, and Maximal Correlation. | Several datasets were utilized in the analysis: (1) three types of scatterplots representing different associations—linear, parabolic, or sinusoidal—either without noise or with two levels of added noise; (2) pairs of independent variables; and (3) pairs of correlated variables, which included various types of relationships such as linear, step-function, W-shaped, sinusoidal, circular, or heteroskedastic noise. | Xi is an equitable coefficient, although the concept of equitability requires more precise mathematical definition, and this property should be quantifiable through computational simulations. Additionally, Xi is consistent and includes a permutation test for assessing the significance of correlation. For example, consider a scenario where (X, Y) is generated from a mixture of bivariate normal distributions, resulting in two clusters with significant noise. In this case, MIC approximated a value of 1, suggesting a perfect relationship, while Xi yielded a value of approximately 0.48, which more accurately reflected the noisy nature of the relationship. While MIC is a powerful tool for detecting patterns, its tendency to overestimate the strength of noisy relationships can lead to less reliable measures of dependence in such contexts. | [3] |
| Xi, Spearman's correlation, and the integrated non-parametric test. | Four different correlation patterns were examined to cover both monotonic and non-monotonic relationships: linear, quadratic, sinusoid, and stepwise (with added noise or constant values). | The new test showed satisfactory power across all settings, effectively detecting both monotonic and non-monotonic relationships. The asymptotic p-values were positively biased for small sample sizes, but the bias diminished with larger sample sizes. For small samples, a permutation test is recommended. | [62] |
| MIC values calculated using AppMIC, ChiMIC, and BackMIC algorithms. | 13 pairs of noiseless functional correlations, including: Line, parabolic, cubic, exponential, non-Fourier frequencies (low, medium, high), linear + periodic frequencies (low, medium, high), varying frequencies (low, medium, high). The study also examined noisy linear, parabolic, and sinusoidal correlations. | BackMIC provided more reasonable grid partitions and MIC values for independent and dependent variable pairs. | [30] |
| UIC, MIC, ChiMIC. | **Functional associations**: linear, parabolic, periodic, cubic, sinusoidal, a chirp function; | (1) **Performance on functional associations**. For most functional associations, the UIC performs comparably to MIC and ChiMIC, especially as the sample size increases. The UIC values approach those of | [31] |

| | | | |
|---|---|---|---|
| | **Non-functional associations**: circle, sinusoidal mixture, two lines, randomly distributed points;<br>**Multidimensional associations**: linear, parabolic, cubic, sinusoidal, a multi-frequency sinusoidal relationship, multiple linear relationships, independent variables, spherical, two distinct plate-like structures, two sinusoidal relationships in a multidimensional setting.<br><br>(Noisy non-functional associations, which are similar to the non-functional associations but with added noise drawn from a uniform distribution.) | MIC for larger datasets. The UIC is less effective in capturing high-frequency components in functional associations when the sample size is small, but this difference diminishes as the sample size increases.<br><br>(2) **Performance on non-functional associations**. The UIC generally produces smaller values than MIC for non-functional associations, which helps in reducing false positives. The UIC is more robust in detecting independence between variables, showing lower values for random associations compared to MIC.<br><br>(3) **Multidimensional Variables**. The UIC can be easily extended to multidimensional variables, providing a computationally efficient measure of association in such settings. MIC and ChiMIC are not as effective for multidimensional variables and require multiple runs to measure associations between all pairs of dimensions, making them computationally expensive. | |
| $R^2$, maxCor, dCor, MIC, Xi, HHG, and $R^2_{GU}$, $R^2_{GS.}$ | (1) Eight simulations in which various types of mixtures of linear dependences were tested. (2) Comparisons of n=100 finite Gaussian samples, with asymptotic distributions. | Generalized Pearson correlation can well capture heterogenous linear relationships. In particular, the K-lines algorithm is the most powerful index of correlation when the scatterplot pattern is a mixture of positive and negative linear associations. | [63] |
| PCC, Spearman correlation coefficient, MIC, clustermatch correlation coefficient (CCC). | **Anscombe's quartets**: I, II, III, and IV. **Continuous variables**: random/independent, non-coexistence pattern, quadratic and two-lines patterns.<br>**Categorical variables:** random/independent, clustered categorical variables, clusters formed by numerical and categorical values. | **Overall**. Pearson and Spearman are the fastest to compute, given that they only require basic summary statistics from the data. In particular, Pearson is three orders of magnitude faster than CCC. Among the nonlinear coefficients, CCC is faster than both variations of MIC, with the exception of very small data sizes. CCC allows for easy parallelization, which can further speed up computation. For example, using three CPU cores, CCC was twice as fast as using a single core for a data size of a million. MIC is very computationally intensive, making it impractical for large datasets despite the methodological and implementation improvements.<br><br>**Accuracy**. CCC accurately identified both linear and nonlinear relationships in simulated datasets, outperforming Pearson and Spearman in detecting complex patterns. CCC was robust to outliers and correctly identified no relationship in cases where outliers influenced Pearson and Spearman.<br>**Flexibility**. CCC effectively handled both numerical and categorical variables, providing a high level of flexibility in detecting various types of patterns.<br>**Statistical Significance**. CCC provided statistically significant values for complex patterns that were missed by traditional linear-only coefficients. | [13] |

| | | | |
|---|---|---|---|
| $K_c$, Pearson's correlation, Kendall's tau, and dCor. | (1) The time-course expression of two genes follows the same sine function but with a phase shift of π/6.<br>(2) The time-course expression of two genes follows the same cosine function, but the magnitudes of gene 1 are twice those of gene 2.<br>(3) The time-course expression of two genes follows the same cosine function, but the magnitudes of gene 1 are twice those of gene 2.<br>In each case, there are three noise levels: low, medium, and high. | (1) The study concludes that Kc-RBF is highly effective in detecting nonlinear correlations, particularly in scenarios where the data contains moderate noise. It consistently outperforms traditional correlation measures such as Pearson's r and distance correlation (dCor) in these conditions. Kc with polynomial kernels, however, tends to have high false positive rates, making it less reliable for accurate correlation detection. Similarly, Kendall's tau shows poor performance across all tested scenarios, indicating it is not suitable for estimating nonlinear correlations.<br>(2) Proper selection of the kernel and tuning of its parameters, such as the γ value in the RBF kernel, are crucial for optimizing the performance of Kc. This careful tuning can significantly enhance the ability to detect nonlinear relationships in the data. Overall, Kc-RBF stands out as a robust and effective measure for nonlinear correlation, while Kc with polynomial kernels and Kendall's tau are not recommended due to their limitations. The study highlights the importance of using appropriate methods and parameters to achieve accurate correlation detection in gene expression data. | [7] |

**Supplementary Table S3. Representative Applications of Information-Theoretic Nonlinear Dependence Analysis in Molecular and Omics Research.**

Representative applications of information-theoretic nonlinear dependence measures, particularly maximal information coefficient (MIC)-based frameworks, in transcriptomic, epigenomic, cancer, aging, network-inference, and genetic association studies. The table summarizes analytical objectives, rationale for selecting nonlinear dependence measures, and major biological insights obtained in studies where linear-only association frameworks were insufficient to capture heterogeneous, nonlinear, or non-monotonic molecular relationships. Emphasis is placed on feature selection, co-expression analysis, oscillatory and age-associated molecular dynamics, regulatory-network inference, and complex dependence structures relevant to systems-level omics analysis.

| Application area. | Analytical task. | Reasons for the use of MIC. | Comment. | Reference. |
|---|---|---|---|---|
| Angiogenesis. | Ranking candidate proteins. | The authors suspected that there could be linear, non-linear as well as non-monotonous associations between the concentrations of growth factors and microvessel density (MVD). | Bot PCC and Xi were used to rank angiogenesis-related proteins/peptides in association between their density and MVD. | [64] |
| Cancer research. | Selecting features among explanatory variables to construct well-defined statistical models. | Samples of lung cancers were clustered according to multiplicity of CNVs, and non-linear association was the statistical tool of choice to select informative features. | Only chromosomal locations with significant values of MIC were selected as model features for the classification of tumors. Note that both MIC and normalized mutual information (NMI) were used for feature selection. | [65] |
| | | MIC was applied to select features (*i.e.* gene-targeting probes) on an expression microarray for the problem of cancer classification. | The importance and correlation of features were considered at the same time for the purpose of feature selection using graph feature. | [66] |
| | Calculating a co-expression network. | WGCNA was modified into an alternative algorithm, WGCNA-P+M, which combines PCC and MIC as similarity measures focused on linear, as well as nonlinear correlations between genes, respectively. | Two real datasets, liver hepatocellular carcinoma (identified as TCGA-LIHC) and colon cancer (series GSE44861) were used to calculate co-expression networks and resulting gene modules were analyzed. The method, which combined linear and non-linear correlations, resulted in more biologically meaningful gene modules and identified more cancer genes. | [67] |
| Aging research. | Correlating concentrations of circulating small noncoding RNAs in plasma and serum with age. | A non-linear correlation between levels of the RNA with age was discovered using MIC. Indeed, there were age-related sncRNA in serum, and some varied non-linearly with age. | Some circulating RNAs changed in concentration linearly with age, but this publication focused on non-linear correlation, for example on RNAs rapidly switched on in expression at a certain age. | [68] |

| | | | | |
|---|---|---|---|---|
| | Correlating beta values of methylation markers with age. | The workflow of the analysis was called DICNAP. Note that MIC was calculated, tested and applied along with PCC to highlight both non-linear and linear correlations. | Many associations of methylation markers with aging were discovered, and they seemed frequently non-linear. Datasets can be identified in GEO as GSE87571 (GPL13534), GSE40279 (GPL13534). | [50] |
| Inference of gene regulatory networks (GRNs). | Constructing GRNs using mixed entropy optimizing context-related likelihood mutual information (MEOMI). | MEOMI was benchmarked against eight other kinds of GRN inference algorithms (*e.g.* CLR, GENIE3, ARACNE, CMI2NI, NARROMI, MRNET, PIDC and BiXGBoost) using the popular DREAM challenge dataset. Note that the pairwise gene associations within the GRNs were likely to be either linear, or non-linear, or non-monotone. | Two DREAM challenge simulated datasets were investigated, namely DREAM3 and DREAM5. Moreover, three real Escherichia coli datasets (SOS pathway network, SOS DNA repair network and community network), plus two human datasets were investigated. | [69] |
| Epistasis in genetics. | Identifying epistasis in case-control association studies. This analytical strategy assumed that joint and marginal distributions of SNPs differed in cases of disease versus controls. | Epistasis was detected statistically as deviation from simple additive effects in terms of distribution of alleles at separate loci. Such deviations were detectable using the values of non-linear correlation, MIC, in comparison to the presumed linear trend. Note that there was also a permutation strategy to establish the statistical null distribution for SNP frequencies. | MIC was calculated for pairs of SNPs (pairwise) for all cases and for all controls. Differences between the two MIC values obtained for each SNP was denoted as ΔMIC. This strategy was as good as competing methods at detecting pure epistasis. However, MIC-based strategy was more likely to be affected by linkage disequilibrium. | [70] |
| Molecular evolution. | Investigating gene expression changes during the prenatal-to-postnatal transition in 5 placental mammals: human, rhesus macaque, mouse, rat, and rabbit. | Co-expression networks were created for a total of 8,238 one-to-one orthologs in four of five placental mammals. As many as 1,425 samples were processed for gene expression profiling. Firstly, a WGCNA network was calculated utilizing PCC. Secondly, another co-expression network was calculated using MIC to test the robustness of the PCC-based network. | The toggle switch model largely explained how physiological adaptations occurred during a prenatal-to-postnatal transition. In other words, switching gene expression largely occurred at the perinatal stage. | [71] |

**Supplementary Table S4. Representative Applications of Rank-Based Nonlinear Dependence Analysis in Molecular and Single-Cell Research.**

Representative applications of rank-based nonlinear dependence measures, particularly Chatterjee's correlation coefficient (Xi), in molecular biology, single-cell inference, cancer genomics, developmental trajectories, and genotype–phenotype analysis. The table summarizes analytical scenarios in which rank-based dependence estimation was used to identify monotonic and non-monotonic relationships, characterize heterogeneous differentiation trajectories, quantify treatment-associated latent structure, and improve feature selection in high-dimensional biological datasets. Emphasis is placed on the robustness, scalability, and distribution-free properties of rank-based inference frameworks in modern omics and systems-level biological analysis.

| Application area. | Analytical task. | Reasons for the use of Chatterjee's coefficient. | Comment. | Reference. |
|---|---|---|---|---|
| Cell biology. | Correlating TF expression with pseudo-time within a subtype of T cells to identify TFs important for cell differentiation. | Chatterjee's correlation coefficient could identify genes that were either monotonically or non-monotonically correlated with pseudo-time along the differentiation trajectory. | Non-monotonically correlated TFs had to be identified as they could represent crucial junctions at which cells were committed along the differentiation pathway. | [54] |
| Single-cell methods. | Quantifying whether individual components of ICA correlate with treatments in CINEMA-OT. | It was proposed that confounding factors for a given statistical analysis could be identified and differentiated from treatment effects in ICA, if an ideal statistical test was used to analyze individual components of the data reduction technique. | The authors developed an approach to single-cell perturbation analysis, named CINEMA-OT, in which Xi is used to quantify whether component correlated with a treatment. A component means a fundamental component of biological functioning. Sources of confounding variation include cell-cycle stage, microenvironment, chromatin accessibility.--- | [53] |
| Zoology and genetics. | Exploring the interface between murine chromosomal evolution and phenotypes observed in mice. | Deviation from the standard race was expressed after statistical shape analysis using Procrustes distance. This distance was then correlated with chromosome number. | The authors computed and tested the correlation between the diploid number and Procrustes distance. To identify both linear and non-linear relationships, both PCC and Xi were computed. However, it was noted that Xi is not a symmetric coefficient and it asymptotically on the interval between 0 and 1.--- | [72] |
| Cancer research. | Screening features of a statistical model using a rank-based correlation coefficient. | The authors could identify informative predictors (*i.e.* genes linked to expression values) and their dependence structures. The proposed algorithm gave precise classification as well as accurate prediction on real-world data. | Expression values of preselected genes were good predictors of disease. (Note that other supervised learning methods build predictive models based on the entire expression dataset.) | [73] |

**Supplementary Table S5. Comparative Evaluations of Nonlinear Dependence Measures Using Real-World Biological Datasets.**

Representative comparative studies evaluating nonlinear dependence measures using real-world transcriptomic, multimodal, cancer, and systems-biology datasets. The table summarizes how different inferential frameworks perform under biologically heterogeneous conditions involving oscillatory transcriptional dynamics, nonlinear co-expression structure, multimodal integration, heterogeneous dependence patterns, and high-dimensional molecular association analysis. Emphasis is placed on comparative inferential sensitivity, biological interpretability, robustness to complex nonlinear structure, and the ability of different methods to identify biologically meaningful associations beyond conventional linear correlation frameworks.

| Indexes benchmarked | Dataset | Goal | Conclusions | Reference |
|---|---|---|---|---|
| Xi, MIC, and Maximal Correlation. | Yeast transcripts whose expression levels were measured over the course of the cell cycle [63]. | Selecting genes that exhibit oscillatory expression patterns across 23 consecutive time points in the time-course. | The Xi correlation outperformed other indices in selecting genes that exhibited true oscillatory transcription patterns, as other indices tended to prioritize monotonic trends over cyclical behaviors. Specifically, Xi identified 215 genes that were not detected by other methods, such as MIC, distance correlation, HHG, and HSIC. Among these, the top six genes identified exclusively by Xi exhibited near-perfect oscillatory behavior. Additionally, a random sample of six genes from the 215 identified by Xi also displayed strong oscillatory patterns, further demonstrating that the performance of Xi was consistent and reliable across the dataset. In contrast, the genes identified by other tests but not by Xi typically exhibited slight increasing or decreasing trends, or heteroscedasticity, without clear oscillatory behavior. This underscores that Xi is more sensitive to detecting cyclical patterns in gene expression data. | [3] |
| Xi, Spearman's correlation, and the integrated non-parametric test. | Yeast transcripts whose expression levels were measured over the course of the cell cycle [63]. | Selecting genes that exhibit oscillatory expression patterns across 23 consecutive time points in the time-course. | The integrated test identified 734 significant genes whose expression levels change during the cell cycle, compared to 619 genes identified by Spearman's test and 385 genes identified by Chatterjee's test. This demonstrates the increased sensitivity of the new test in detecting significant genes. | [74] |
| $R^2$, maxCor, dCor, MIC, Xi, HHG, and $R^2_{GU}$, $R^2_{GS}$. | Expression levels of 26 genes involved in the glucosinolate biosynthesis pathway were measured across 232 samples, considering various conditions, treatments, replicates, and tissue types. | To calculate the correlation in expression between pairs of genes within the target pathway, as compared to other genes. | The K-lines algorithm not only clusters genes associated with the glucosinolate pathway but also partitions datasets based on subtypes of the tissue of origin [64]. | [63] |

| | | | | |
|---|---|---|---|---|
| UIC, MIC, ChiMIC. | A dataset sourced from the World Health Organization (WHO), which includes potential factors influencing life expectancy, with a particular focus on developed countries over the five-year period from 2010 to 2015. Associations examined in this dataset are: (1) Life expectancy versus GDP per capita; (2) Life expectancy versus government health expenditure. | 1. Analyzing the relationship between life expectancy and GDP per capita in developed countries. 2. Analyzing the relationship between life expectancy and government health expenditure as a percentage of total government expenditure in developed countries. | In the analysis of the WHO dataset, the UIC identified a stronger association between life expectancy and government health expenditure than between life expectancy and GDP per capita, which was consistent with the observed data patterns. In contrast, MIC and ChiMIC methods did not differentiate as distinctly between these associations. | [31] |
| PCC, Spearman correlation coefficient, MIC, clustermatch correlation coefficient (CCC). | Human Gene Expression Data from the Genotype-Tissue Expression (GTEx) Project: This dataset specifically includes GTEx v8 data across various tissues, with a primary focus on RNA-seq data from whole blood, encompassing 755 samples. | The top 5,000 genes exhibiting the largest variance in whole blood were selected for analysis. Pairwise similarity matrices for these 5,000 genes were then computed using various correlation coefficients, including Pearson, Spearman, MIC, and CCC. | The distribution of CCC values is more skewed compared to Pearson and Spearman, suggesting that CCC is capable of capturing a broader range of relationships, including non-linear patterns. CCC values were found to be more similar to Spearman than to Pearson. CCC demonstrated a high degree of agreement with either Pearson or Spearman in 90.5% of gene pairs, assigning either a high or low correlation value consistently. Over 20,000 gene pairs that were highly ranked by CCC were not similarly ranked by the other correlation coefficients, highlighting CCC's capacity to identify unique relationship patterns. Gene pairs ranked highly by CCC in the GTEx whole blood dataset showed stronger replication in independent, tissue-specific networks from the GIANT project. CCC-ranked gene pairs were more likely to be associated with the same biological processes and were predicted to be specifically expressed in blood cell lineages. | [13] |
| Pearson, Spearman, dCov, HHG, and MIC. | The CNV dataset used in the study was obtained from a panel of 60 human cancer cell lines known as the NCI-60. The dataset includes comparative genomic hybridization (CGH) data, which originally contained 349 clones. After excluding clones with missing values and those with unknown gene symbols, the final analysis was performed on a set of 99 CGH clones, representing 99 genes. The | The goal of this comparative analysis is to evaluate the effectiveness and equitability of various nonlinear correlation measures in detecting complex relationships in large datasets. By comparing these methods, the analysis aims to identify the most robust and computationally efficient tools for uncovering significant associations in molecular sciences and other high-dimensional data contexts. | (1) Univariate Analysis: The HHG test detected twice as many associations as traditional tests, especially non-linear and non-monotone ones.<br><br>(2) Multivariate Analysis: dCov and HHG tests effectively linked genes of unknown function to cancer-related pathways, offering deeper biological insights.<br><br>(3) Combined Approach: Using both univariate and multivariate tests provides a comprehensive analysis, uncovering many associations missed by traditional methods, aiding in understanding complex gene networks in cancer. | [75] |

| | | | | |
|---|---|---|---|---|
| | data was used to test all possible pair-wise associations among these 99 genes, generating 4851 pairs for analysis. | | | |
| Kc, PCC, dCor, Kendall's Tau. | (1) RNA-seq data of the early phase of Th17 cell differentiation and T-cell activation (Th0) cells: This dataset includes gene expression profiles from Th0 and Th17 cells at 0, 12, 24, 48, and 72 hours, with three replicates for each gene.<br><br>(2) Time-course microarray gene expression data of yeast cell cycle genes: This dataset includes gene expression profiles of six gene pairs involved in the yeast cell cycle, collected at 18-time points over two cell cycles. | The goal of analyzing these datasets is to identify significant nonlinear correlations in gene expression:<br><br>(1) Th17 cell differentiation: to uncover genes involved in early Th17 cell differentiation by identifying those with significant nonlinear correlations with IL17A.<br><br>(2) Yeast cell cycle: to estimate nonlinear correlations between cell cycle genes in yeast, providing insights into cell cycle regulation and its implications for disease studies. | The conclusions from analyzing these real-world datasets are:<br><br>(1) Th17 Cell Differentiation: The Kernelized correlation (Kc) method successfully identified four genes (TIAM1, ISG20, RAB3, and RORC) with significant nonlinear correlations with IL17A, indicating their involvement in early Th17 cell differentiation. This method outperformed other techniques like DESeq and distance correlation (dCor).<br><br>(2) Yeast Cell Cycle: Kc effectively detected nonlinear correlations in gene pairs involved in yeast cell cycle regulation, including both similar and complementary patterns. It outperformed Pearson's correlation and dCor, particularly in identifying negative correlations. | [7] |

**Supplementary Table S6. Representative Methods for Oscillatory and Temporal Dependence Analysis.**

Representative nonlinear approaches for rhythmic, oscillatory, and temporal dependence inference in molecular time-series data.

| Method | Analytical principle | Key strengths | Main limitations | Representative applications |
|---|---|---|---|---|
| MICOP | Template-based MIC optimization | Sensitive detection of oscillatory structure | Dependent on waveform specification | Circadian transcriptomics |
| JTK_CYCLE | Rank-based periodicity testing | Robust rhythmicity detection | Reduced sensitivity to irregular oscillations | Time-series gene expression |
| Cosine regression | Harmonic model fitting | Interpretable periodic inference | Assumes sinusoidal structure | Circadian and temporal omics |
| Gaussian-process temporal models | Probabilistic temporal inference | Handles irregular sampling | High computational cost | Longitudinal transcriptomics |
| Kernel-based temporal inference | RKHS-based local dependence estimation | Captures heterogeneous temporal structure | Kernel parameter sensitivity | Single-cell trajectory analysis |
| Rank-based temporal methods | Ordinal temporal dependence estimation | Robust under sparse sampling | Reduced local sensitivity | Oscillatory transcriptomic screening |

**Supplementary Table S7. Extended Practical Decision Framework for Nonlinear Dependence Analysis in Molecular and Omics Research.**

Extended scenario-oriented decision framework summarizing recommended nonlinear dependence measures according to analytical objective, sample size, dependence structure, computational scalability, and implementation constraints in molecular and omics data analysis. The table expands the main-text practical guidance framework by incorporating inferential rationale, methodological trade-offs, major analytical limitations, and workflow-level implementation recommendations relevant to transcriptomic screening, oscillatory and temporal inference, feature selection, mixed-type data integration, and large-scale systems biology applications.

| Scenario | Objective | Recommended methods | Rationale | Limitations | Implementation notes |
|---|---|---|---|---|---|
| Small sample size ($n < 50$) | To detect dependence reliably when estimator variance and bias are high due to limited observations. | Chatterjee's Xi and the TICe estimator are recommended as primary methods. | Rank-based methods such as Xi provide stable estimates because they do not rely on density estimation, while TICe offers strong statistical power for independence testing even in small datasets. | Mutual information–based methods such as MIC may produce inflated dependence estimates due to positive bias in small samples, and kernel-based methods may become unstable without careful parameter tuning. | For very small datasets ($n < 30$), permutation-based significance testing should be used to ensure reliable inference and avoid reliance on asymptotic approximations. |
| Large-scale screening ($10^5$–$10^6$ variable pairs) | To efficiently identify statistically significant associations while controlling computational cost and multiple testing burden. | A two-stage procedure should be used in which TICe, UIC, or Xi is first applied to filter candidate associations, followed by MICe to rank the remaining relationships. | Filtering methods such as TICe and Xi provide high statistical power and computational efficiency, while MICe enables equitable ranking of the strongest associations across diverse functional forms. | Exhaustive computation of MIC across all variable pairs is computationally prohibitive, and kernel-based approaches may become infeasible due to quadratic memory requirements. | The filtering stage should be used to reduce the number of candidate pairs before applying more computationally intensive ranking methods, thereby improving scalability and reducing false discovery burden. |
| Time-series and oscillatory data | To detect non-monotonic and periodic relationships that are not captured by linear or monotonic measures. | Chatterjee's Xi, MICOP (when a reference waveform is known), and combined Xi–Spearman approaches are recommended. | Rank-based methods such as Xi are sensitive to oscillatory and non-monotonic dependence patterns, while MICOP enhances detection when a biologically meaningful reference waveform is available. | MICOP performance depends strongly on the choice of reference waveform, and mutual information–based methods may exhibit upward bias in small-sample time-series data. | When the underlying waveform is unknown or irregular, Xi should be preferred due to its robustness and lack of parametric assumptions. |
| Feature selection for classification | To identify predictive variables while minimizing overfitting and ensuring stability of selected features. | TICe should be used to filter candidate features, followed by MICe for ranking, with optional integration of linear and nonlinear measures. | Combining filtering and ranking methods balances statistical power and equitability, while integrating linear and nonlinear measures improves robustness across different relationship types. | Reliance on a single nonlinear measure may lead to overfitting, particularly in high-dimensional, low-sample settings, and mutual information estimates may be biased in sparse data. | Feature selection should be combined with cross-validation or stability analysis to ensure that selected variables generalize beyond the training dataset. |

| | | | | | |
|---|---|---|---|---|---|
| Mixed numerical and categorical data | To measure dependence in datasets that include both continuous and categorical variables without introducing encoding bias. | The Clustermatch Correlation Coefficient (CCC) and Chatterjee’s Xi are recommended. | Clustering-based methods such as CCC can directly compare partition structures across variable types, while rank-based methods such as Xi remain invariant under monotonic transformations. | CCC performance depends on the quality and stability of clustering, and MIC-based methods may perform poorly when categorical variables produce sparse grid partitions. | Clustering parameters should be carefully selected and validated, and sensitivity analysis is recommended to ensure robustness of detected relationships. |

**Supplementary Table S8. Representative Single-Cell and Spatial Omics Applications of Nonlinear Dependence Analysis.**

Representative nonlinear dependence frameworks used in single-cell, spatial, and multimodal omics workflows.

| Methodological framework | Representative application | Inferential objective | Main advantages | Key limitations |
|---|---|---|---|---|
| Kernel-based manifold inference | Single-cell trajectory reconstruction | Local nonlinear structure detection | Sensitive to manifold geometry | Kernel parameter dependence |
| Graph-aware neighborhood inference | Cell-state and neighborhood modeling | Context-aware regulatory inference | Integrates cellular topology | Reduced interpretability |
| Distance-based multimodal integration | Cross-modal association analysis | Detection of heterogeneous multimodal structure | Distribution-free inference | Matrix scaling |
| Gaussian copula integration | Single-cell multi-omics | Dependence estimation independent of marginals | Handles mixed distributions | Computational complexity |
| Graph neural network integration | Regulatory network reconstruction | Higher-order systems inference | Integrates multimodal neighborhood structure | High infrastructure requirements |
| Pseudotime-aware nonlinear inference | Dynamic trajectory analysis | Temporal regulatory inference | Models heterogeneous developmental structure | Pseudotime uncertainty |

**Supplementary Table S9. Representative Software Ecosystems and Computational Frameworks for Nonlinear Dependence Analysis.**

Representative software implementations and computational environments used in nonlinear dependence analysis workflows.

| Software ecosystem / framework | Methodological focus | Representative algorithms or methods | Programming environment | Representative capabilities | Common applications | Scalability profile | Key methodological considerations |
|---|---|---|---|---|---|---|---|
| minerva | Information-theoretic | MIC, MICe, TICe | R | Model-free nonlinear dependence screening | Transcriptomic and epigenomic association analysis | Moderate | Adaptive partitioning improves flexibility but increases computational burden |
| MICtools | Information-theoretic pipeline | TICe, MICe | Python | Parallelizable filtering and ranking workflow | Large-scale omics screening | High | TICe filtering improves scalability for high-dimensional datasets |
| MIC implementations | Information-theoretic | ApproxMaxMI (Approx-MIC) | Java / R / Python | Equitable nonlinear dependence estimation | General nonlinear association analysis | Low | Computationally intensive dynamic-programming grid search |
| ChiMIC | Information-theoretic | ChiMIC | MATLAB / Python | $\chi^2$-based optimized partition estimation | Large transcriptomic datasets | Moderate | Improved efficiency may reduce flexibility for complex oscillatory patterns |
| BackMIC | Information-theoretic | BackMIC | MATLAB | Backward grid-search optimization | Noisy nonlinear association analysis | Moderate | Improved partition accuracy but still computationally intensive |
| UIC implementations | Information-theoretic | UIC | MATLAB / Python | Uniform partition-based nonlinear screening | High-dimensional exploratory analysis | High | Uniform partitioning improves speed but may miss fine-grained nonlinear structure |
| XICOR | Rank-based | Chatterjee's Xi | R / Python | Distribution-free scalable dependence estimation | Large-scale transcriptomic screening | High | Efficient and robust but less sensitive to weak smooth nonlinear structure |
| energy | Distance-based | Distance correlation (dCor) | R | Distance covariance and independence testing | Multimodal omics integration | Moderate | Matrix scaling limits extremely large datasets |
| copula | Copula-based | Gaussian and semiparametric copulas | R | Copula modeling and dependence estimation | Mixed-type omics analysis | Moderate | High-dimensional optimization and covariance regularization required |
| huge | Graphical modeling | Sparse graphical modeling | R | High-dimensional graph estimation | Network reconstruction | High | Requires regularization tuning in sparse graph estimation |

| | | | | | | | |
|---|---|---|---|---|---|---|---|
| kernlab | Kernel-based | Kernelized correlation (Kc) and kernel methods | R | Kernel-based statistical learning | Single-cell and multimodal analysis | Moderate | Performance depends strongly on kernel selection and parameter tuning |
| scikit-learn | Kernel and graph learning | Kernel methods and graph learning | Python | Machine-learning infrastructure for nonlinear inference | General nonlinear inference workflows | High | General-purpose infrastructure requiring workflow customization |
| CCC implementations | Clustering-based | Clustermatch Correlation Coefficient (CCC) | Python | Clustering-based dependence estimation | Mixed-type and subgroup-specific co-expression analysis | High | Sensitive to clustering quality and noise structure |
| Scanpy | Graph-/network-aware | Neighborhood graph inference | Python | Neighborhood and manifold analysis | Single-cell transcriptomics | High | Scalability depends on graph-construction complexity |
| PyTorch Geometric | Graph neural networks | Graph neural network inference | Python | GPU-accelerated graph learning | Network-aware multimodal inference | GPU-accelerated | Requires substantial computational infrastructure |
| Nextflow | Workflow management | Workflow orchestration | Platform-independent | Reproducible pipeline execution | Large-scale omics workflows | Distributed | Improves reproducibility and computational portability |
| Snakemake | Workflow management | Workflow orchestration | Python | Scalable workflow automation | Transcriptomic and multimodal analysis | Distributed | Supports reproducible large-scale computational workflows |
| Docker / Singularity | Containerization | Portable computational environments | Platform-independent | Containerized reproducible inference | Reproducible nonlinear dependence analysis | Workflow-oriented | Facilitates reproducibility across heterogeneous computing environments |
